# *In vitro* genotoxicity evaluation and metabolic study of residual glutaraldehyde in animal-derived biomaterials

**DOI:** 10.1093/rb/rbaa041

**Published:** 2020-10-24

**Authors:** Jianfeng Shi, Huan Lian, Yuanli Huang, Danmei Zhao, Han Wang, Chunren Wang, Jingli Li, Linnan Ke

**Affiliations:** Institute for Medical Devices Control, National Institutes for Food and Drug Control, No. 31 Huatuo Road, Beijing 102629, China

**Keywords:** glutaraldehyde, cytochrome P450, biocompatibility evaluation of medical devices, animal-derived biomaterials

## Abstract

Glutaraldehyde (GA) is an important additive that is mainly used in animal-derived biomaterials to improve their mechanical and antimicrobial capacities. However, GA chemical toxicity and the metabolic mechanism remain relatively unknown. Therefore, residual GA has always been a major health risk consideration for animal-derived medical devices. In this study, extracts of three bio-patches were tested via the GA determination test and mouse lymphoma assay (MLA). The results showed that dissolved GA was a potential mutagen, which could induce significant cytotoxic and mutagenic effects in mouse lymphoma cells. These toxic reactions were relieved by the S9 metabolic activation (MA) system. Furthermore, we confirmed that GA concentration decreased and glutaric acid was generated during the catalytic process. We revealed GA could be oxidized via cytochrome P450 which was the main metabolic factor of S9. We found that even though GA was possibly responsible for positive reactions of animal-derived biomaterials’ biocompatibility evaluation, it may not represent the real situation occurring in human bodies, owing to the presence of various detoxification mechanisms including the S9 system. Overall, in order to achieve a general balance between risk management and practical application, rational decisions based on comprehensive analyses must be considered.

## Introduction

Glutaraldehyde (GA, CAS Registry No. 111-30-8), a cross-linking agent, is widely used in medical devices derived from tissues, such as bio-patches, bio-prosthesis and bio-valves. The incorporation of GA can improve the biomaterials’ mechanical integrity and long-term durability, reduce degradation and preserve sterility [1]. One major drawback of GA is its potentially toxic effects toward recipients exposed to residues and/or chemicals released from a reverse cross-linking reaction. Thus, residual toxicity of GA cross-linked biomaterials has become of critical importance when considering potential clinical applications [2, 3].

The toxicology of GA has been extensively studied over the last three decades. However, among the reports variations in the genotoxicity of GA results is evident. GA has been intensively evaluated using numerous *in vitro* and *in vivo* systems. Although *in vivo* genotoxicity studies have shown negative results [4–6], variable results have been achieved for *in vitro* studies, including bacterial tests and mammalian cell tests. In the case of bacterial reverse mutation test system, GA has displayed weak mutagenic activity toward *Salmonella typhimurium* and *Escherichia coli* [4, 7–10]. In the mammalian cell lines test system, the results are not consistent. For Chinese hamster ovary (CHO) studies, gene mutation of hypoxanthine–guanine–phosphoribosyl transferase (HGPRT) shows negative results [4], with sister chromatid exchanges tests displaying different results, even in inter-laboratory comparison [11]. Compared with chromosomal aberration, DNA mutation can be detected by both mouse lymphoma assay (MLA) and TK6 assay [12, 13]. The genetic damage detectability of MLA is more sensitive than a chromosomal aberration, and the viable cells capable of forming colonies created more objectionable results [14].

We found that mainly the appearance of metabolic activation (MA) system’s affects MLA tests. MLA test results are positive only in the absence of MA (un-MA) [12, 13]. The Organization for Economic Co-operation and Development (OECD) guidelines suggest that mammalian liver post-mitochondrial fraction (S9) has been generally used as a metabolic activator [15]. The S9 system comprises of a variety of ubiquitously distributed enzymes and plays an essential role in drug metabolism. The main metabolic factor of S9 is cytochrome P450 (CYP) isozyme, which is defined as the monooxygenases that responsible for the oxidative metabolism of drugs and environmental chemicals [16]. CYP3A4, part of the CYP family, is a subset that is the highest expressed in the liver [17] and responsible for the bulk of chemical agents. Nicotinamide adenine dinucleotide phosphate (NADPH) is required in the CYP catalytic system by transferring electrons [18]. Previous reports show that the treatment of stimulated polymorphonuclear leukocytes with GA can effectively maintain a higher and longer activated state of NADPH oxidase under the inactive conditions, such as high temperature, high concentration of NaCl and positively charged alkylamine [19]. This indicates that GA may have some connection with NADPH oxidase.

GA is an important additive in the manufacturing of animal-derived biomaterials, but its toxicity must be considered. Compared with the conflicting results of GA from different *in vitro* models, *in vivo* tests show consistent negative results. Additionally, a difference remains between the system with or without an MA in MLA. Whether the metabolic activation plays an essential role, it is worthy of further investigation. Although the function of CYPs has been well investigated, the interaction between CYPs and GA has yet to be reported.

The aim of the present study was to investigate both the potential release of GA from biological patches and its genotoxic potential *in vitro* on MLA. The S9 effect on GA was also investigated further.

## Materials and methods

### Biological patch

The patches were purchased from three corporations. All patches did not obtain the approval of the National Medical Products Administration (NMPA) of China. Confidentiality was maintained throughout this work, the companies’ information was not published.

### Cell

Heterozygous L5178Y TK ± 3.7.2C cells were obtained from the Chinese National drug safety assessment and monitoring center. The cells were routinely grown in RPMI1640 medium (HyClone). The growth medium was supplemented with 10% heat-inactivated horse serum (Gibco), 100 U/ml penicillin and streptomycin (HyClone).

### S9

S9 was purchased from Tianjin Institute of Medical Sciences. The protein content and efficiency was verified by that department.

### Cytochrome P450 CYP3A4 isozyme with cytochrome P450 reductase

CYP3A4 was purchased from Sigma.

### Chemicals and reagents

Acetonitrile [high-performance liquid chromatography (HPLC) grade], used as mobile phases in HPLC, was purchased from Fisher Chemical. 2,4-Dinitrophenylhydrazine was obtained from Sinopharm Chemical Reagent Co. Ltd. Perchloric acid was obtained from Shanghai Jinlu. Formic acid was obtained from Fisher Chemical. Acetonitrile (hypergrade for LC-MS), used as mobile phases in LC-MS, was purchased from Merck. Glutaraldehyde solution (50%) was purchased from Sigma. Glutaric acid was purchased from Shanghai Alddin Biochemical Technology Co. Ltd. Methanol was purchased from Fisher Chemical.

### Preparation of extracts from samples

Three animal-derived biomaterials were involved. The extraction conditions and methods were conducted per *ISO 10993-12:2012*. Before the extracting treatment, the cleaning procedure was performed. After cleaning with 0.9% sodium chloride injection, 120 rpm for 5 min, three times, each sample was extracted in 0.9% sodium chloride injection at 37°C for 72 h and the extract ratio was 3 cm^2^/ml.

### HPLC determination of GA

HPLC method was used for determining GA concentration, which used derivatization of 2,4-dinitrophenylhydrazine to bis-2,4-dinitrophenylhydrazone. The chromatographic conditions were based on the GA determination method of biological products [20]. The column was CAPCELL PAK C_18_ column (250 × 4.5 mm 5 μm), with acetonitrile: H_2_O (70:30) mobile phase at a flow rate of 1.2 ml/min. The injection volume was 10 μl and UV detection was at 360 nm. The DNPH solution was prepared in 30% aqueous perchloric acid. Standard solutions with which to calculate curves were prepared by stepwise dilution with the deionized water (DI) ranging in concentration from 1 to 10 μg/ml for GA. A 500 μl sample or standard solutions were mixed with 500 μl mobile phase and 50 μl of the DNPH solution. Then the prepared solutions were subjected to HPLC (Agilent 1260) analysis. GA concentration (μg/ml) of the samples was determined using a calibration graph. If the GA concentration of the samples did not fit well with the calibration graph, it should be further diluted with DI.

### Ultra-performance liquid chromatography-mass spectrometer determination of glutaric acid

#### Sample extraction

The samples were extracted using solid-phase extraction (SPE) employing Oasis^®^ MAX 3 cc/60 mg Cartridge (P/N 186000367). A 500 μl aliquot of sample was diluted with 1 ml 2% NH_4_OH (pH = 10) and loaded onto an SPE cartridge previously conditioned with 2 ml methanol and equilibrated with 2 ml water separately. Samples were eluted with 500 μl 2% FA in methanol and transferred to an auto-sampler vial for ultra-performance liquid chromatography-mass spectrometer (UPLC-MS/MS) analysis.

#### UPLC-MS/MS assay

Prepared stock standard solution with a glutaric acid concentration of 1000 mg/l in DI water. Working standard solution 1 mg/l was freshly prepared by diluting the stock solution with DI water. The analysis was performed on a SHIMADZU UPLC-MS/MS 8050 System. The chromatographic separations were performed using a HALO C18 (100 mm × 2.1 mm, 2.7 μm) column with a mobile phase of 0.1% FA in acetonitrile: 0.1% FA in water (20:80) at a flow rate of 0.3 ml/min. The injection volume was 1 μl. Column temperature: 40°C. The column effluent was monitored using electrospray ionization in the negative mode with multiple reactions monitoring (MRM). The transition 131.10 > 87.00 was employed for glutaric acid. (For glutaric acid confirmation; for glutaric acid quantification).

### Mouse lymphoma assay

The spontaneously occurring TK^−/−^ cells were periodically cleaned. S9 combined with cofactors and culture medium to form the metabolic activation system, the volume ratio of S9 was 1%. MLA was performed according to OECD guideline 490. The volume ratio of the test extract was 10% in the final treatment medium.

### The inactivation of S9 and validation of inactivated efficiency

S9 was placed in a water bath at 56°C for an hour, and then the inactivated S9 and normal S9 were used as MA factors in the *Salmonella Typhimurium* Reverse Mutation Test (AMES Test) of 2-aminofluorene. AMES Test was performed according to OECD guideline 471. The catalytic effect was evaluated and recorded.

### Determination of GA in different reaction system

Inactivated S9 was added with cofactors, which was grouped as in the MA system. The cofactors consisted of 200 μM NADP^+^, 250 μM glucose-6-phosphate, 1.65 mM K^+^ and 400 μM Mg^2+^. In the group of MA, S9 was added into the system with cofactors and 1% volume ratio. In the group of in-MA and un-MA, inactive S9 and 0.9% sodium chloride injection were added, respectively, and 1% volume ratio. In each group, the initial concentration of GA was 2 μg/ml. After the reaction, GA concentration was determined by HPLC at 30 min, 1 h, 2 h and 4 h.

### Determination of GA and glutaric acid in metabolic system

The metabolic system was performed at 30°C, 120 r/min. The cofactors consisted of 200 μM NADP^+^, 250 μM glucose-6-phosphate, 1.65 mM K^+^ and 400 μM Mg^2+^. The S9 assay system consisted of 150 μg/ml GA, 0.1 ml S9 and cofactors in phosphate buffer. The CYP assay system consisted of 150 μg/ml GA, 80 μM CYP3A4 with reductase and cofactors in phosphate buffer. The observation time point was 30 min. GA concentration was determined by HPLC, and glutaric acid was determined by UPLC-MS.

### Statistical analysis

The data were expressed as mean ± standard deviation. The statistics were calculated with the SPSS version 13.0 for Windows (SPSS, Chicago, IL, USA). Differences between groups were compared using either two-tailed Student’s *t*-test or analysis of variance. Differences were considered significant at *P < 0.05 and **P < 0.01.

## Results

### The residual GA determination and MLA test of animal-derived biomaterials

**Figure 1.**
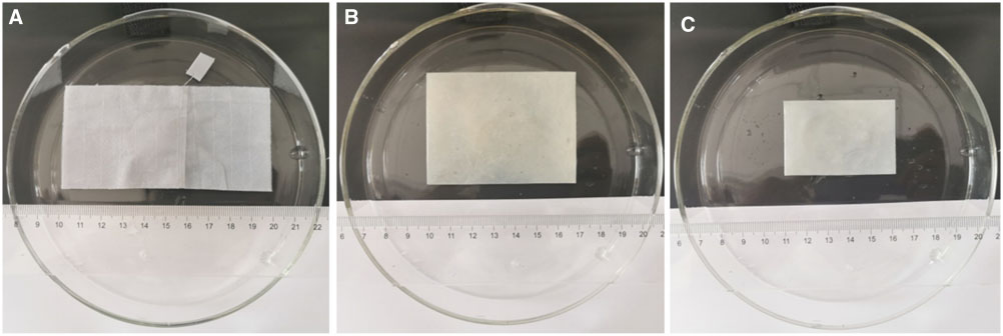
Pictures of the prepared biological patches.

Three biological patches were prepared (Fig. 1), and GA concentrations from three extracts were determined by HPLC. The results show that GA concentration differs widely among the three samples, which suggests that the dissolving level of residual GA is affected by the manufacturing process (Table 1). MLA test shows that the MA system has a great influence on the final result. The sample extracts were genotoxic or cytotoxic without the MA, and no genotoxic with MA. In the case of sample A, which has the lowest GA concentration of 20 μg/ml, promoted the positive reaction in the un-MA system. Above 30 μg/ml GA concentration the relative survival of cells is <10%, which corresponds to higher cytotoxicity. According to OECD guideline 490, the obtained results could not be evaluated (Table 1).

**Table 1.** GA Concentration and MLA test results of sample extracts

Test sample (*n* = 4)	CONC. (μg/ml) mean(±SD)	MLA result	
		Without MA	With MA
A	20.03 (1.57)	P	N
B	37.07 (1.54)	T	N
C	30.39 (3.63)	T	N

T, toxic (The cytotoxicity was too severe, and the relative survival is less than 10%, below the acceptability criteria.); P, positive; N, negative.

### MLA results of GA solution

To validate if the positive results of MLA were related to GA concentration, various concentrations of GA solutions were examined. According to the GA concentration of sample A, 24 μg/ml of GA was chosen as the maximum dose level, and 3, 6, 12 μg/ml as the lower dose. GA was diluted in 0.9% sodium chloride injection and the volume ratio of GA was 10% (v/v) in the final treatment medium. In the absence of MA, positive MLA results are observed and GA produced dose-dependent effects at 3 μg/ml GA concentration (Table 2). Interestingly, all samples in the MA system show negative results. Nevertheless, the representation of cell survival values, such as relative suspension growth (RSG) and relative total growth (RTG), are higher. The cytotoxicity and genotoxicity results of GA can be relieved by the S9 metabolism.

**Table 2.** Test results of GA solution in MLA

	CONC. (μg/ml)	PE_0_ (%)	PE_2_ (%)	RSG (%)	RTG (%)	MF (×10^−6^)
Without MA	Control	70	76	100.0	100.0	91.9
	0.3	57	62	81.0	66.3	156.7
	0.6	57	59	63.3	49.0	227.3^a^
	1.2	28	37	31.4	15.3	435.0^a^
	2.4^b^	1	1	1.5	1.3	800.0^a^
	MMS	30	34	80.0	45.6	544.9^a^
With MA	Control	88	94	100.0	100.0	100.8
	0.3	87	101	105.1	113.8	77.8
	0.6	80	68	119.6	86.5	153.4
	1.2	74	72	92.8	71.1	105.7
	2.4	13	53	17.7	10.1	201.3
	CTX	17	16	35.9	8.6	738.3^a^

PE, plate efficiency; MF, mutation frequency.

Positive control: VMMS: methyl methanesulfonate, 10 μg/ml; CTX, cyclophosphamide, 6 μg/ml.

a Positive result.

b The cytotoxicity of 2.4 μg/ml GA was too severe, and the relative survival was <10%, below the acceptability criteria.

c The experiment was repeated once and consistent result was obtained.

### The S9’s effect on GA concentration

**Figure 2.**
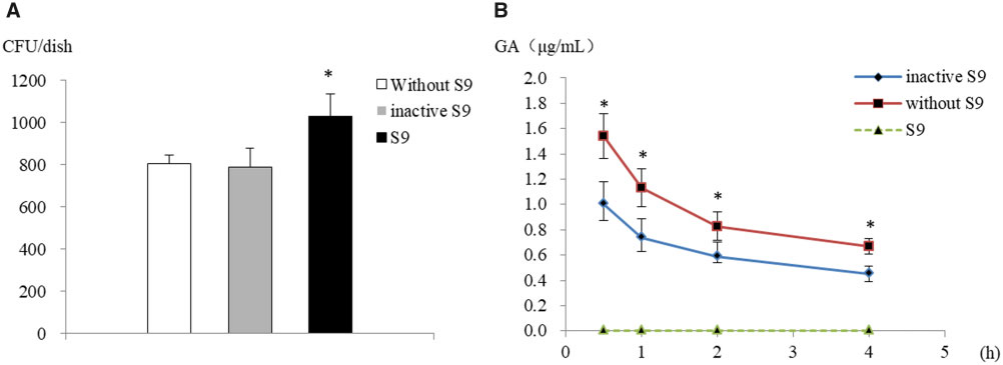
The affected of GA concentration in different reaction systems. (**A**) S9 was inactivated by water bath heating. For inactive S9 group, CFU value was close to blank and showed a significant decrease compared with normal S9. (**B**) The green dotted line represents GA in S9 metabolic system; the red and blue full line represent GA in blank and inactive metabolic system, respectively. In S9 metabolic system, GA shows a rapid decline below the detection limit after at 30 min. For inactive S9 system, GA significantly decreases compared with blank. Due to the reaction system containing amino acids, which can crosslink with GA, over prolonged time GA concentration decreases. CFU, colony forming units. Mean ± SD, *n* = 3.

We performed three reaction systems, including blank, inactive S9 and S9, to investigate S9’s effect. Inactive S9 was obtained by heat-inactivation, and the effect was validated by the AMES test system (Fig. 2A). In the S9 metabolic system, GA was not detected due to the concentration being beyond the limit of detection. The concentration of GA shows a significant decrease in the inactive S9 system compared with the S9 blank system at each time point (Fig. 2B). We hypothesized that owing to the protein crosslinking character of GA, it might contribute to GA consumption. This was demonstrated by the decrease in GA concentration with the metabolic activation of S9, in which the P450-NADPH pathway may play an essential role.

### Metabolite detection of GA in S9 catalyst system

**Figure 3.**
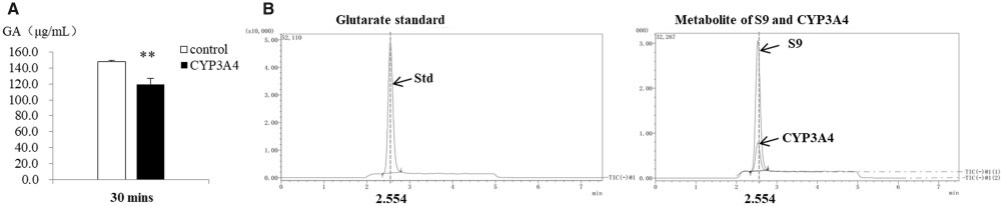
Oxidation of GA via P450 pathway, and the generation of glutaric acid in the metabolic system. (**A**) GA in CYP3A4 metabolic system shows a significant decline compared with blank. Mean ± SD, *n* = 3. (**B**) Total Ion Current (TIC) chromatograms of glutaric acid standard and metabolite in S9 and CYP3A4 metabolic system. Glutaric acid was eluted at 2.554 min retention time. Quantification was accomplished by selected ion monitoring (SIM) using ions corresponding to each of the dicarboxylic acids at m/z 131 for glutaric acid. As S9 was obtained from rat liver homogenate with more complexity than recombinant CYP3A4, it was very difficult to accurate the species and concentration of CYP family members in S9. The cofactors concentrations were equal in both reaction systems, but CYP concentrations differ in both systems. In this case, there was an obvious difference of metabolite concentration between S9 and CYP3A4 reaction systems.

In order to investigate if GA could metabolize via the P450-NADPH pathway, excess GA was added into the CYP3A4 catalytic system. After 30 min incubation, GA concentration shows a significant decrease in CYP3A4 group (Fig. 3A). The major function of CYPs is oxygen activation, in which the hydroxylation reaction is the key factor [21]. Therefore, it can be assumed that glutaric acid may be the metabolite. Analysis of the metabolite by UPLC-MS/MS revealed peaks at ∼2.5 min corresponding to the deprotonated molecular ions [M-H]- (m/z 130.9) for glutaric acid. Glutaric acid can be identified by retention time and characteristic fragments at m/z 87.00 and 112.90, respectively. The characteristic fragment ion at m/z 87.0 caused by in-source collision induced dissociation (CID), and corresponds to the loss of one carboxyl group using a cone voltage setting of 60 V. Both S9 and CYP3A4 metabolic system displayed the generation of glutaric acid (Fig. 3B).

## Discussion

In the evaluation of animal-derived biomaterials, the toxicity of samples was detected via *in vitro* models, such as cytotoxicity or genotoxicity tests. Through analysis of the samples’ manufacture procedures, it was found that they were all treated with GA. Previous reports have described the obvious cytotoxicity of GA treated bovine pericardium [22]. In our study, we demonstrated that GA-crosslinked bio-patches released considerable amounts of GA, which could cause a positive reaction of MLA, as well as displaying toxic effects on cell viability. Additionally, the toxicity of GA was obvious at a microgram degree in the evaluations of cytotoxicity and genotoxicity. The genotoxicity of GA was verified in previous studies through the application of the MLA model [12]; however, the toxic relief of GA in MA system was of interest. MLA results showed that the GA concentration was significantly decreased in the presence of S9, promoting relief of cytotoxicity and genotoxicity.

The results also showed that that the *in vivo* studies possessed particular resistance of organisms to GA compared with *in vitro* models. There were several possible mechanisms to account for this finding. Studies have revealed that the detoxification effect is related to metabolism and protein-binding [23], however, such mechanisms were not investigated further. In this work, we hypothesized that metabolism was more effective than protein-binding. The major metabolite of GA is CO_2_ [6] with the majority of GA being metabolized in the first 4 h [24]. In previous studies, the proposed metabolism involves the oxidation of GA to glutaric acid, which can further metabolize to CO_2_ via coenzyme A [25]. The mechanism of glutaric acid metabolism has been well documented, but how GA metabolizes to glutaric acid is not well known. We firstly verified that GA could metabolize to glutaric acid via CYP catalysis and proposed a novel pathway. On the one hand, CYPs are mainly expressed in the liver, and little in the kidney [26]. On the other hand, the kidney and liver are the cumulative organs of GA [27]. It has been reported that certain chemical agents can undergo oxidization by CYPs, which can then be cleared and excreted by the kidney [28]. Therefore, we inferred that the liver and kidney play a leading role in the storage and detoxification of GA. Furthermore, the rapid metabolism of GA makes it hard to reach a cumulative toxicity in the body. However, the released GA can accumulate in the kidney and liver, its toxicity may negatively impact the function of those organs. Toxicological studies of GA via inhalational and oral administrations were widely conducted. There is no pathology change in the kidney and liver when the animals are exposed to GA via body inhalation at 62.5–1000 ppb for 13 weeks [29]. Rats accepted an administration of 0.25% GA in drinking water for 11 weeks, with no pathologic injury being detected in the kidney and liver [25]. Compared with the two exposure routes, few systemic toxicological studies focus on the exposure route via direct or indirect contact with the blood. In view of the above situation, the risk assessment procedure that closer to the application of animal-derived biomaterials should be considered.

In tissue engineering products, GA has been applied as a protein crosslinking and antimicrobial agent for more than 50 years [30]. However, GA not only brings about product revenue but also a safety risk. The products present positive results in *in vitro* models if GA is not cleaned efficiently. In contrast, the mechanical and antibacterial superiorities can be sacrificed if we merely apply a lower concentration of GA. Animal-derived biomaterials are currently used mainly in blood-contacting medical devices, whereas GA has less toxicology data in that route of exposure. In a study that examined the toxicology data of the oral route, rats were chosen as the animal model and the no observed adverse effect level (NOAEL) of GA was 5 mg/kg/day BW [31]. According to ISO 10993-17:2009, the tolerable daily intake (TDI) value can be calculated from NOAEL and modifying factor (MF), the calculation formula is:

(1) $$\mathrm{TDI}=\frac{\mathrm{NOAEL}}{\mathrm{MF}}\;,$$

where

MF is UF1×UF2×UF3.

UF1 accounts for inter-individual variation among humans;

UF2 accounts for extrapolation from data derived in a species other than humans; UF3 accounts for the quality and relevance of the experimental data.

In most cases, MF between 10 and 1000 is sufficiently protective. AT MF of 1000, a higher coefficient is observed, where TDI of GA is 0.005 mg/kg/day. Furthermore, the tolerable exposure (TE) can be calculated from TDI, body mass (m) and utilization factor (UTF).

(2) TE=TDI×m×UTF 

(3) UTF=CEF×PEF ,

where

CEF is a concomitant exposure factor. According to *ISO* *10993-17*: *2009*, CEF can be ‘0.2’ if the utilization factor is unknown.

PEF is proportional exposure factor, PEF = *n*_exp_/*n*_use._ (The *n*_exp_ is the number of days in the exposure category; *n*_use_ is the number of days of device use). The animal-derived medical device is long-term implanted product. Therefore, the PEF value is 1.

From these data, UTF value is 1.

When normal body mass is 70 kg, TE value of GA in the human body is 0.07 mg/day. Importantly, this is only a rough calculation based on the international standard and limited systemic toxicity data; however, more data and sensitive methods are required.

The determination of GA toxicity is a big challenge for biocompatible evaluation of tissue engineering biomaterials. In this work, we demonstrate the genotoxicity and cytotoxicity of GA in *in vitro* model. But, we also found the relief phenomenon in the metabolic system suggesting that the positive results of *in vitro* evaluation model did not entirely represent the condition *in vivo*. In the future, bio-responsive risk represented by positive results should be evaluated in order to gauge the balance between the risk and income of these medical devices. Establishing the accepted standard of GA would benefit the animal-derived medical device supervision and development.

## Conclusion

1. The release of GA in animal-derived biomaterials could induce significant cytotoxic and mutagenic effects in mouse lymphoma cells.
2. In the presence of the S9 metabolic system, the GA toxicity was attenuated, indicating that GA could metabolize via the P450-NADPH pathway, generating glutaric acid.
